# Using beta binomials to estimate classification uncertainty for ensemble models

**DOI:** 10.1186/1758-2946-6-34

**Published:** 2014-06-22

**Authors:** Robert D Clark, Wenkel Liang, Adam C Lee, Michael S Lawless, Robert Fraczkiewicz, Marvin Waldman

**Affiliations:** 1 Department of Life Sciences, Simulations Plus, Inc., 45205 10th Street West, Lancaster, CA 93534, USA

**Keywords:** Artificial neural network ensemble, ANNE, Classification, Confidence, Error estimation, Predictive value, QSAR, Uncertainty

## Abstract

**Background:**

Quantitative structure-activity (QSAR) models have enormous potential for reducing drug discovery and development costs as well as the need for animal testing. Great strides have been made in estimating their overall reliability, but to fully realize that potential, researchers and regulators need to know how confident they can be in individual predictions.

**Results:**

Submodels in an ensemble model which have been trained on different subsets of a shared training pool represent multiple samples of the model space, and the degree of agreement among them contains information on the reliability of ensemble predictions. For artificial neural network ensembles (ANNEs) using two different methods for determining ensemble classification – one using vote tallies and the other averaging individual network outputs – we have found that the distribution of predictions across positive vote tallies can be reasonably well-modeled as a beta binomial distribution, as can the distribution of errors. Together, these two distributions can be used to estimate the probability that a given predictive classification will be in error. Large data sets comprised of logP, Ames mutagenicity, and CYP2D6 inhibition data are used to illustrate and validate the method. The distributions of predictions and errors for the training pool accurately predicted the distribution of predictions and errors for large external validation sets, even when the number of positive and negative examples in the training pool were not balanced. Moreover, the likelihood of a given compound being prospectively misclassified as a function of the degree of consensus between networks in the ensemble could in most cases be estimated accurately from the fitted beta binomial distributions for the training pool.

**Conclusions:**

Confidence in an individual predictive classification by an ensemble model can be accurately assessed by examining the distributions of predictions and errors as a function of the degree of agreement among the constituent submodels. Further, ensemble uncertainty estimation can often be improved by adjusting the voting or classification threshold based on the parameters of the error distribution. Finally, the profiles for models whose predictive uncertainty estimates are not reliable provide clues to that effect without the need for comparison to an external test set.

## Background

Drug discovery and development is an expensive business and its costs continue to rise. Exploitation of quantitative structure activity relationships (QSARs) and related *in silico* methods have the potential to speed development and reduce costs considerably, and regulatory agencies have expressed support for doing so [1-3]. Considerable progress has been made in recent years on assessing the overall predictive reliability of QSAR models, but research and regulatory applications both require good ways to estimate the accuracy of *individual* predictions. Considerable work has been done on ways to identify compounds for which predictions are unlikely to be reliable – i.e., on applicability domains [4-6] and on quantitative estimations of uncertainty for regression models [4,7-13]. Some prior work has made use of ensemble variance for categorical estimation of confidence [14,15]. To our knowledge, however, the degree of ensemble consensus in terms of votes has not been utilized to make quantitative estimates of predictive classification uncertainty for individual predictions.

A key first step on the path to successfully exploiting ensemble variance for classification error analysis was to move away from the traditional truth table assessment of classification performance based on four classes of predictive outcome – true positives, true negatives, false positives and false negatives – and to look instead at distributions of predictions and errors as a function of the level of consensus among the individual submodels making up the ensemble. In particular, we investigated how predictive classification error rates (errors/prediction) relate to the number of positive “votes” obtained from an ensemble model composed of artificial neural networks [16] – e.g., classification ANNEs generated by the ADMET Modeler™ module of ADMET Predictor™ – each of which is trained on a different subset of a shared training pool. If each network were fully independent and had the same probability *p* of misclassifying a compound, the contingent probability *P* that *k* of *K* networks in an ensemble will mistakenly assign a “negative” compound to the “positive” class or a “positive” compound to the “negative class” would be expected to follow a binomial distribution:

(1) $$P\left(k|K,p\right)=\left(\begin{array}{l} K\\ k \end{array}\right)p^{k}{\left(1-p\right)}^{K-k}$$

Such a scheme was explored (data not shown), but proved unsatisfactory. In retrospect, this is not surprising, since the networks are not independent and have different probabilities of making an erroneous prediction for different compounds. When such “overdisperse” distributions are encountered in biometrics problems, researchers have found beta binomial distributions (*BB*) useful [17-19]:

(2) $$\mathrm{BB}\left(k|K,\alpha ,\beta \right)=\left(\begin{array}{l} K\\ k \end{array}\right)\frac{B\left(k+\alpha ,K-k+\beta \right)}{B\left(\alpha ,\beta \right)}$$

Here, *B* is the beta function:

(3) $$B\left(\alpha ,\beta \right)=\frac{\Gamma \left(\alpha \right)\Gamma \left(\beta \right)}{\Gamma \left(\alpha +\beta \right)}$$

where $\Gamma \left(x\right)$ is the gamma function [20], which is a continuous extension of the factorial (*Γ*(*n* + 1) = *n* !). Unlike the binomial distribution, the beta binomial can be convex as well as concave. The former is the case for α < 1 and β < 1 whereas the latter holds for α > 1 and β > 1. When α = β = 1, the beta binomial distribution reduces to the discrete uniform distribution on the interval 0 to *K*.

We attempted to fit beta binomial distributions to uncertainty profiles (i.e., the misclassification or error rate as a function of the number of *K* networks making *k* positive votes for a given compound) directly but the results were not satisfactory. On the other hand, separate beta binomials fit distributions of ensemble predictions and error counts for the training pool reasonably well. This is not altogether surprising, since both distributions result from a series of *K* events that are related but not independent. There are two possible outcomes in both cases: “the compound in question is a positive or a negative” for predictions and “the prediction is correct or incorrect” for errors. What is somewhat surprising is that the distributions fitted to the training pool match the corresponding distributions seen for large external validation sets as well.

The distributions of votes and errors can then be combined to yield an ensemble uncertainty profile using a method rooted in elementary probability theory. Let *P(ϵ|k)* be the probability that a given prediction is in error given that it receives *k* positive votes, which we equate with the predictive uncertainty of the classification. Let *P(k|ϵ)* be the probability that a prediction receives *k* positive votes *given* that it is erroneous (misclassified). Finally, let *P(ϵ)* be the overall probability that a prediction is in error and let *P(k)* represent the probability that a prediction receives *k* positive votes. Application of Bayes’ theorem yields:

(4) $$P\left(\epsilon |k\right)P\left(k\right)=P\left(k|\epsilon \right)P\left(\epsilon \right)$$

To distinguish between underlying population distributions and estimated distributions fitted to samples, we introduce the following notation. Let *φ*(*k*) represent our estimate of *P(k)*, the probability distribution of positive vote tallies across all predictions or simply the “prediction distribution”. Let ϵ(*k*) represent our estimate of *P(k|ϵ)*, the probability distribution of all misclassified predictions as a function of *k,* which we refer to as the “error distribution”. *P(ϵ|k)* is the probability of an error in classification (i.e., the uncertainty of a prediction) that receives *k* positive votes; our estimate of it will be represented by *u(k)* and referred to as the uncertainty profile or distribution.

Finally, *P(ϵ)* is the overall probability of an error or simply the misclassification rate (*MR*) of the model. Our estimate of the uncertainty of a prediction receiving *k* votes is then given by:

(5) $$u\left(k\right)=\mathrm{MR}*\epsilon \left(k\right)/\phi \left(k\right)$$

*MR* is obtained from the overall misclassification rate (i.e., overall number of incorrect predictions divided by the total number of predictions) for the training pool, and ϵ(*k*) and *φ*(*k*) are estimated by fitting beta binomial distributions to the training pool errors and predictions, respectively, as functions of *k*.

Our protocol for vote-based ensemble classifications can be summarized by the following:

1. Build an ensemble of *K* submodels.

2. Establish a classification threshold for each submodel that determines its vote.

3. Tally the number of positive votes *k* for each prediction.

4. Establish a decision rule for the ensemble.

5. Classify each ensemble prediction for the training pool as being correct or incorrect.

6. Count the number of errors for each vote tally *k.*

7. Add a continuity correction to the count for each tally by adding 1 to each prediction tally and adding 0.5 to each error count.

8. Find the alpha and beta parameters of a beta binomial distribution, *φ*(*k*), that best matches the cumulative voting distribution as a function of *k* by minimizing the Kolmogorov-Smirnov (K-S) statistic [21] between the observed and beta binomial distributions.

9. Find the optimal beta binomial distribution, ϵ(*k*), that best matches the observed distribution of the errors by similarly minimizing the K-S statistic between the cumulative distributions.

10. Estimate the uncertainty distribution as *u*(*k*) = MR*ϵ(*k*)/*φ*(*k*).

The remainder of this paper is devoted to showing applications of this protocol to data sets of varying quality and showing the influence of imbalances in class size. Most of the examples described involve models where the ensemble decision is determined by voting: decision thresholds are established independently for each network and the classification “votes” that result are tallied to determine the ensemble classification. For unbalanced data sets, resetting the ensemble voting threshold to match the mean of ϵ(*k*) may substantially improve the overall balance between specificity and sensitivity.

One application makes use of an alternative method in which individual network outputs are averaged and compared to an aggregate classification threshold. In such cases, the model benefits from resetting the threshold to the geometric mean of the averaged threshold and the mean of ϵ(*k*). Working with large validation sets proved essential to getting good enough sampling of errors to meaningfully assess how well our uncertainty estimation method works. Typically, the numbers of predictions and errors receiving *k* votes is small for intermediate values of *k* (i.e., those not near the extremes of 0 and *K*), which can result in low counts in this region unless the validation set is comprised of thousands of compounds. This is especially true for good models. The desire to maximize the size of the validation set to minimize the effects of noise in assessing the performance of the model led us to use an unusually small fraction of the available data to train the models – 10 to 30% instead of the typical 80 to 90%. It also helped to demonstrate that the methodology does not require large training sets to work.

Note that though the examples examined here all involve artificial neural network ensembles, the algorithm above is cast in more general terms: we expect the method to be applicable to any ensemble of reasonably robust submodels, regardless of their source.

## Results and discussion

### Balanced data sets

S+logP is the ANNE regression model for octanol:water partition coefficient provided in ADMET Predictor [22]. Its excellent performance in third-party evaluations [23,24] reflects the high quality of the large (12,580 compound), heavily curated data set upon which it is based. For our first example, that data set was split roughly in half for classification purposes by categorizing compounds having log P ≥ 2.0 as “positive” and those having log P < 2.0 as “negative”. Doing so yielded data set “logP2”, which was comprised of 5946 positives and 6634 negatives. The bulk (90%) of the data was set aside for use as an external validation set and was not used or referred to in any way for model building.

The remaining 10% of the data set was used to create artificial neural network ensemble classification models (ANNEs), each made up of 33 individual networks. All networks in an ensemble have the same architecture (same number of neurons and descriptor inputs) but are trained with different subsets of the shared training pool. Details of the model construction are provided in the Methods section, but they are unlikely to affect the general phenomena and conclusions discussed herein.

**Figure 1:**
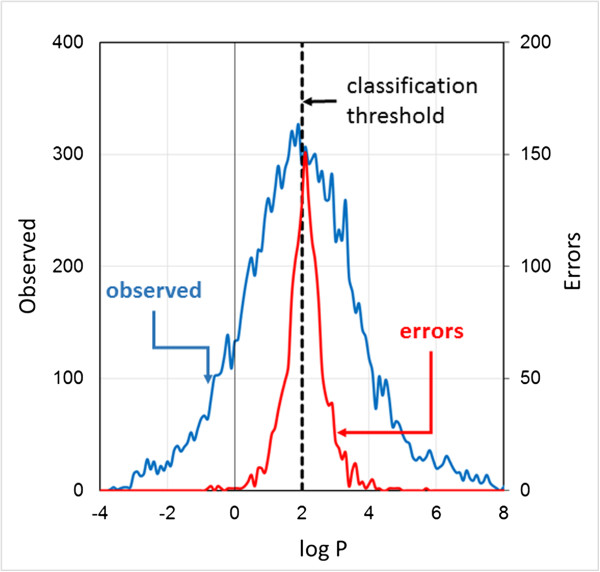
**Distribution of classification errors for the logP2 data set.** Observed logP values for compounds in the data set were binned at intervals of 0.1 and counts within each bin are represented by the blue curve. Data for compounds with logP values below -4 are omitted for clarity, as are those above 8. The distribution of logP values for compounds misclassified by model logP2-1 is shown in red. Note that the scale for error counts is given at the right of the figure and is magnified by a factor of 2.

Figure 1 compares the distribution of erroneous predictions in the target space (i.e., as a function of logP values) with the entire logP distribution for one such model, logP2-1. The error scale at the right in Figure 1 is doubled (relative to the scale on the left) to highlight the fact that the uncertainty is very close to 0.5 at the assignment threshold of logP = 2.0; the model is simply guessing at that point. The number of misclassified compounds falls off sharply for logP to either side of that threshold. Indeed, there is a finite experimental uncertainty (generally about 0.2 in the vicinity of observations for which logP ≈ 2) in the experimental determination of logP, so some of the nominal misclassifications are for compounds that are actually miscategorized in the data set itself. A “false negative” with a data set entry for logP of 2.03, for example, may have a “true” logP value of 1.98, which would make it a *false* false negative. There will, however, be a similar number of cases in which a compound miscategorized in the data set is misclassified by the model, so the overall uncertainty is close to 0.5 at the threshold itself.

The distribution of errors is only roughly symmetrical in this case, in part because the distribution of observed logP values is not quite symmetrical and in part because the positive examples appear to be slightly harder to learn. This is reflected in the difference between the validation set sensitivity (0.849) and specificity (0.882) shown for model logP2-1 in Table 1. The former is the fraction of positives correctly classified as such, whereas the latter is the fraction of negatives classified correctly.

**Table 1:** **Performance statistics for the models described here and their beta binomial parameters**^**a**^

**Data set**	**Model**	**Architect.**^**b**^	**Threshold**	**Sensitivity**	**Specificity**	***J***^***c***^	**α**	**β**	**MR**^**d**^
logP2	1	33×6×40	16.5	0.849	0.882	0.731	0.695^c^	0.772	0.083
	2	33×3×45	16.5	0.861	0.857	0.718	0.635	0.571	0.087
Ames	1	33×2×26	16.5	0.791	0.596	0.387	0.926	0.537	0.222
	2	33×4×24	16.5	0.700	0.676	0.376	0.489	0.462	0.239
logP3	1a	33×4×44	16.5	0.882	0.892	0.774	1.229	0.469	0.077
	1b	33×4×44	24.5	0.840	0.921	0.761	0.925	0.323	0.066
	2a	75×4×42	37.5	0.889	0.885	0.775	1.163	0.472	0.089
	2b	75x4x42	53.5	0.858	0.910	0.768	1.037	0.415	0.083
CYP2D6	1a	33×3×35	16.5	0.721	0.789	0.510	1.561	0.447	0.211
	1b	33×3×35	27.5	0.604	0.873	0.476	1.350	0.294	0.164
logP3	**3a**^**e**^	**33×3×24**^**e**^	16.2	0.874	0.862	0.736	0.891	0.263	0.095
	**3b**	**33×3×24**	20.3	0.862	0.874	0.742	0.690	0.306	0.096

^a^Performance statistics are for compounds in the validation set. Beta binomial parameters shown are those obtained by fitting to the distribution of training pool misclassifications. ^b^“Architect”. indicates the network architecture, given as networks × neurons × inputs. ^c^*J* is Youden’s index [28], which is equal to sensitivity + specificity – 1. ^d^“MR” is the misclassification rate for the training pool. ^e^**Boldface** names and architectures indicate models in which ensemble classifications were determined using the averaging method.

**Figure 2:**
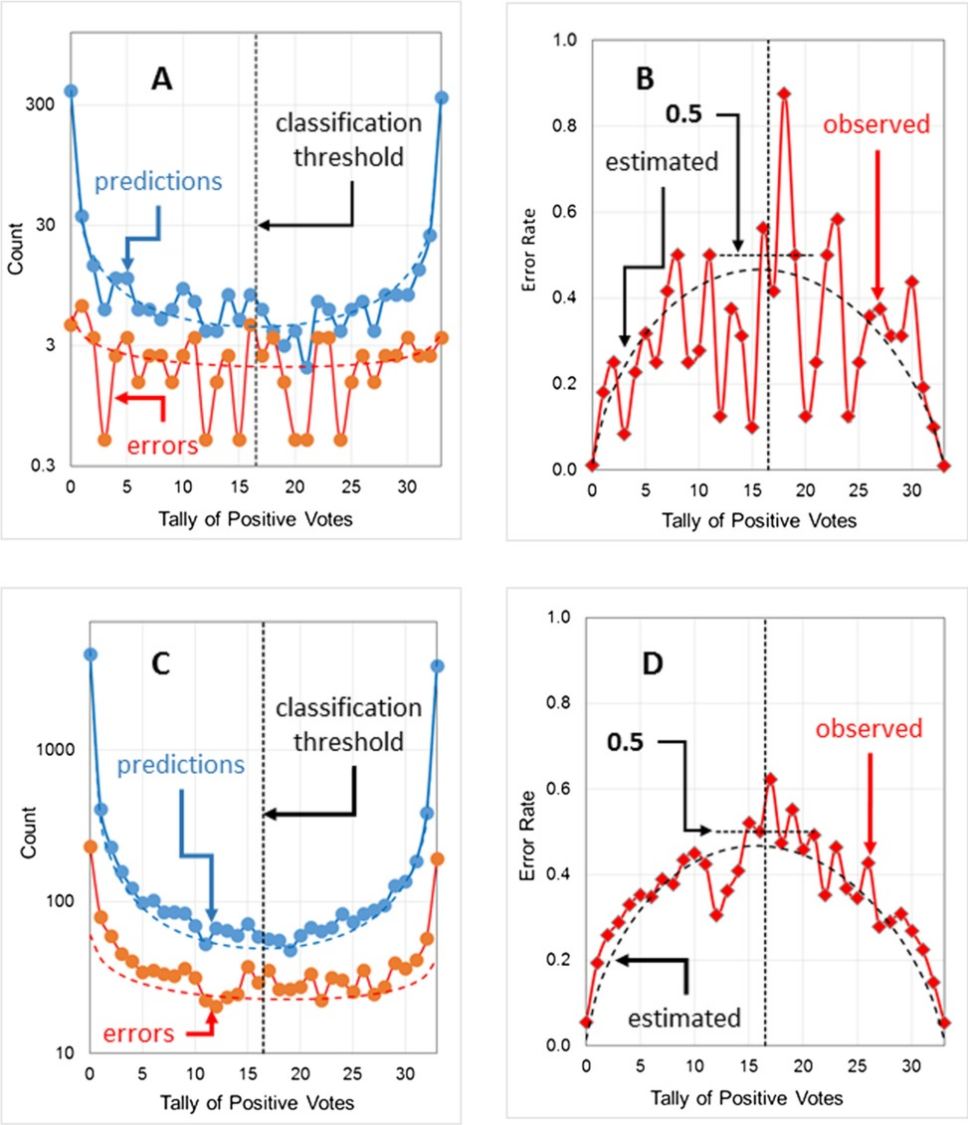
**Estimating the uncertainty profile for the logP2 data set.** The model shown (logP2-1) has six hidden neurons and uses 40 structural descriptors as input. The voting thresholds (indicated by the vertical black dotted lines) was 16.5. The horizontal dotted lines running across the thresholds indicate where an error rate of 0.5 would fall. Error counts include a continuity correction of 0.5 (see text for details). **(A)** Distribution of predictions (blue) and errors (red) for the training pool, with fitted beta binomial distributions shown as dashed lines. **(B)** Distribution of observed error rates (red symbols) for the training pool and the uncertainty calculated from the fitted prediction and error beta binomials (dashed black line). **(C)** Distribution of predictions (blue) and errors (red) for the external validation set. Dashed lines represent the fitted beta binomial distributions for the corresponding training pool results, scaled to account for the larger size (10x) of the validation set. **(D)** Observed (red symbols) error rate profile for the validation set and uncertainty profile (dashed black line) estimated using the beta binomials fitted to the training pool.

Figure 2A shows the distribution of predictions and errors as a function of the tally of positive network votes along with curves for the corresponding fitted beta binomial distributions. The default (naïve) voting threshold used to determine the output classification is simple majority rule: if more than half of the network votes are positive (*k* > 16.5), the compound in question is classified as a positive; otherwise, it is classified as a negative. True and false negatives lie to the left of the threshold, whereas true and false positives lie to the right of the threshold. Figure 2B shows the observed error rate along with the estimated uncertainty profile calculated from the fitted beta binomials in Figure 2A; it is not fitted to the observed error rate profile.There are several things to note about Figure 2A. The first is that the scale on the vertical axis is logarithmic. Most networks agree on most predictions and, where they all agree, the consensus prediction is correct nearly all of the time. Though most errors lie at the extremes of the plot where ensemble consensus is high, the number of predictions there is even higher, so the uncertainty (error rate) is low.

Secondly, the data are very noisy at intermediate values of *k* because the sampling rate is low. The standard error of a rare event count is equal to its square root, so the relative standard error is large for counts below 10. As a result of the noisy data and low sampling counts, all that can be claimed for the training pool is that the uncertainty profile calculated from the training pool prediction and error beta binomials is consistent with what is actually observed. Note, too, that this “calculated uncertainty profile” crosses the ensemble voting threshold just below the theoretical threshold error rate of 0.5 (indicated by the horizontal dotted line in Figure 2B).

Finally, a continuity correction of 0.5 [25] has been applied to all error counts in Figure 2. This addresses a fundamental disconnect: the counts are integers, whereas the beta binomial probabilities to which we are fitting them can take on any value between 0 and 1. Hence uncorrected counts from finite samples can lead to poor estimates for the actual distribution parameters, especially when the expected number of errors (*ϵ*(*k*)*N) for a particular value of *k* is low. The error rate when there is only one prediction, for example, is 0.0 if the prediction in question is correct and 1.0 if it is not, and neither proportion is likely to be a good estimate of the uncertainty. Indeed, in the extreme case where there are no training pool predictions at all – erroneous or otherwise – the uncorrected error rate is equal to 0/0, which is indeterminate. This problem is often encountered when estimating frequencies of rare events, and adding a continuity correction of 0.5 is a standard way to deal with it [25]. A complementary continuity correction of 1 was added to each prediction count, yielding a sample error rate of 0.5 for values of *k* for which the training pool contains no predictions at all.

Figures 2C and D show the distribution of predictions and errors and of error rates for compounds in the external *validation set* along with the beta binomial distributions and uncertainty profiles generated using the distribution parameters derived from fitting to the *training pool* results. The deviations observed are small in both figures, remarkably so for the validation set’s error rate profile (Figure 2D). That the error rate for the validation set tracks the uncertainty profile calculated from the training pool data more closely than the training pool’s own error rate tracks it is due in large part to the validation set having ten times as many observations, which significantly reduces the noise. Note that the observed error rate is again quite close to 0.5 at the ensemble voting threshold of 16.5 (Figure 2D).

**Figure 3:**
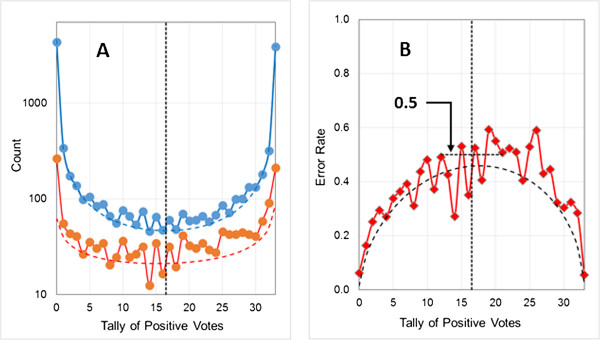
**Variability of calculated uncertainty profiles.** Model logP2-2 uses three hidden neurons and 45 descriptors as input. The voting threshold (indicated by the vertical black dotted line) was 16.5. The horizontal dotted lines running across the thresholds indicate where an error rate of 0.5 would fall. **(A)** Distribution of predictions (blue) and errors (red) for the external validation set. Dashed lines represent the fitted beta binomial distributions for the corresponding training pool results. **(B)** Observed (red symbols) error rate profile for the validation set and uncertainty profile (dashed black curve) calculated from the prediction and error distributions fitted to the training pool.

Figure 3 shows validation set profiles for a different model (logP2-2). This model was generated using a different random number seed, which results in different training pool splits and different initial neural network weights. Not surprisingly, validation set performance statistics for the two models are similar, although the network architectures differ (Table 1). Note, however, that the specificity for logP2-2 is slightly lower than that for logP2-1 (0.857 vs. 0.882; Table 1), which is reflected in more false positives in the test set (errors to the right of the voting threshold of 16.5) for the former. Its observed error rate profile is shifted slightly to the right, just as the lowered value of β shifts the predicted uncertainty profile to the right (Figure 3B vs Figure 2D).

The Ames mutagenicity data set (taken from the publicly available compilation by Hansen et al. [26]) represents a more “real world” classification problem than the logP2 data set. The authors compiled it with admirable care, but the data set can at best be categorized as medium quality, given that variability between laboratories limits the reproducibility of the underlying assay to only about 85% [26,27]. Here, too negatives are roughly balanced with positives and about 10% of the data set was allocated to the training pool.

**Figure 4:**
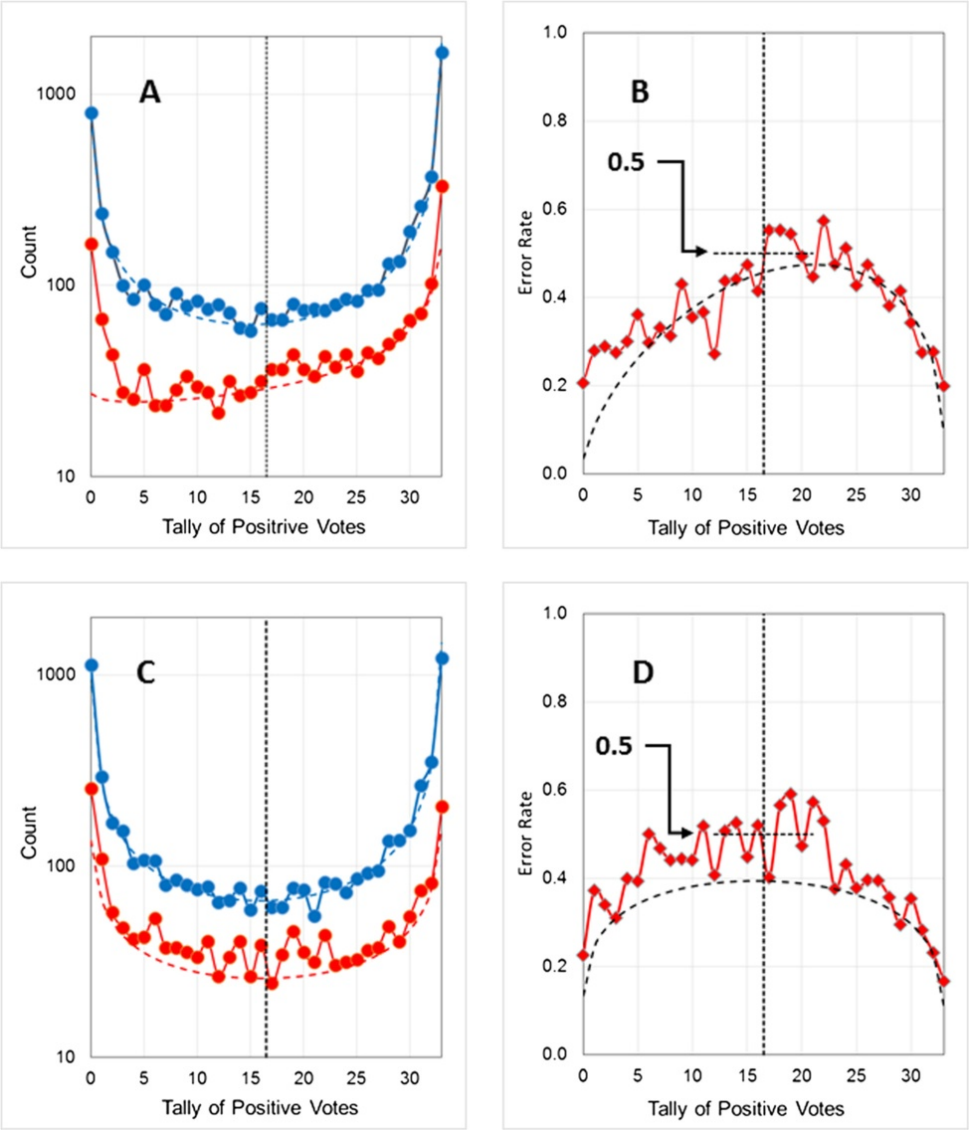
**Estimating the uncertainty profile for the Ames data set.** The validation set results shown in **panels A** and **B** are for a model (Ames-1) having two hidden neurons and 26 descriptors as input, whereas the results shown in **panels C** and **D** are for a model (Ames-2) having four hidden neurons and taking 24 descriptors as input. Different random number seeds were used to split the respective training pools into training and test sets and to initialize the individual ANN weights. The voting threshold for both (indicated by vertical black dotted lines) was 16.5. **(A and C)** Distribution of predictions (blue) and errors (red) for the external validation set. Dashed lines represent the fitted beta binomial distributions obtained from the corresponding training pool results. **(B and D)** Observed (red line) error rate profile for the validation set and predicted uncertainty profile (dashed black line) based on the training pool.

Figure 4 shows results for two models – Ames-1 (Figure 4A and B) and Ames-2 (Figure 4C and D) – obtained with different random number seeds. The statistics for both (Table 1) are inferior to the model performances reported by Hansen *et al.*[26], but that is to be expected given the much smaller training set used here. Nevertheless, estimating the reliability of confidence estimates for relatively weak models is perhaps an even more critical need than making such estimates for models with strong performance statistics.

Ames-1 does a good job of predicting uncertainties at intermediate vote tallies, but underestimates errors somewhat at the extremes. Having some errors at the extremes is consistent with the rather modest historical accuracy rate of 85% for the Ames assay. The fact that most or all of the networks agree on how these compounds should be classified, however, suggests that some of them may be miscategorized due to mistakes in the literature occurring in the course of publication and subsequent data compilation – i.e., that a number of the misclassifications may in fact be *false* false negatives. We have found confidence analysis of the sort described here a valuable tool for identifying such potential errors in data sets.

Ames-2 does a better job of matching the observed error rates at the extremes, but consistently underestimates them (and therefore overestimates confidence in the predictions) at intermediate levels of consensus. These may well be compounds that are borderline in their mutagenicity, but the practical outcome with respect to the reliability of confidence estimates is the same: the profile is less useful than it could be for confidence estimation.Fortunately, Ames-2 provides a clue to its weakness even in the absence of any external validation set, in that its calculated uncertainty profile crosses the voting threshold well below 0.5 – below 0.4, in fact (Figure 4D). To appreciate why this is indicative of a problem, consider a compound receiving a number of positive votes just above or below the voting threshold. A small change in the properties reflected by the model descriptors could flip it from being correctly classified to being misclassified. There has to be a point between the extremes where the threshold is perfectly placed, but as that point is approached, the prediction becomes a guess (at best) and the expected uncertainty will converge to 0.5 (or higher) at the threshold. The fact that the predicted uncertainties at the voting threshold are reasonably close to 0.5 for Ames-1 (Figure 4B) and for the logP2 models described above (Figures 2 and 3) is consistent with their confidence estimations being reliable.The central flattening evident in Figure 4D, in contrast, should be taken as a warning flag. The associated model itself (as opposed to the uncertainty estimate) need not necessarily be discarded, however, especially if it is a case of the profile being distorted by undersampling of the error distribution. When this occurs, it may be preferable to provide aggregate class confidences – i.e., positive and negative predictive values – in lieu of individual confidence estimates.

### Unbalanced data sets

Data sets in which there are many more observations in one class than another are problematic for many classification methods. Rather than trying to balance the training pool (e.g., by undersampling the larger class), ADMET Modeler addresses such imbalances by scaling the terms in the objective function by class size and by relying on Youden’s index *J*[28] to set classification thresholds (see Methods for details). Doing so provides robust performance statistics across a wide range of data sets, but can complicate confidence estimation. To illustrate, an imbalanced logP data set was created by categorizing compounds having log P ≥ 3.0 as “positive” and those having logP < 3.0 as “negative”. Doing so yielded a high-quality data set (logP3) comprised of 3161 positives and 9419 negatives, by coincidence a ratio just over 1:3. The fraction used as the training pool was increased from 10% to 15% to offset the reduced number of positive examples.

**Figure 5:**
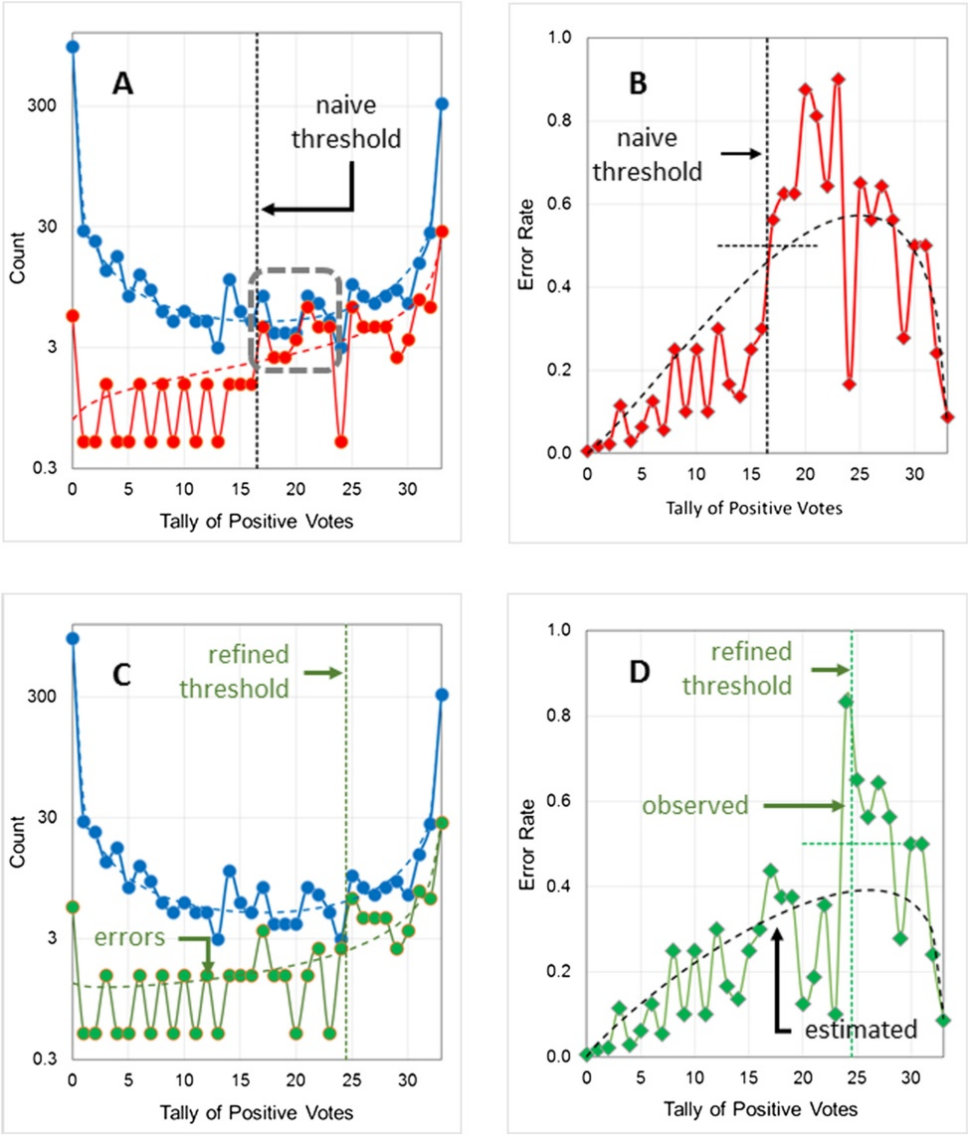
**Adjusting the voting threshold for an unbalanced logP data set.** The vertical dotted lines indicate the applicable voting thresholds. **(A)** Training pool distribution of predictions (blue lines) and errors (red lines) for Model logP3-1a, for which the naïve majority rules threshold of 16.5 was used. Observed distributions are represented by the solid lines and fitted beta binomials by dashed lines. **(B)** Observed and predicted error rate distributions for Model logP3-1a. The red line represents the observed values and the dashed black line represents the predicted profile calculated from the beta binomials shown in panel **A**. **(C)** Training pool distribution of predictions (blue) and errors (green) for Model logP3-1b, for which the voting threshold was shifted to 24.5. Observed distributions are represented by solid lines and fitted beta binomials by dashed lines. **(D)** Observed and predicted training set error rate distributions for Model logP3-1b. The green line represents the observed values and the dashed black line represents the predicted profile calculated from the fitted beta binomials in **panel C**.

Model logP3-1a was built using 33 networks and the naïve majority-voting rule – i.e., with a voting threshold of 16.5. The results are shown in Figures 5A and B for the training pool prediction and error distributions along with their fitted beta binomial distributions. There is a notable stretch of tallies to the right of the threshold (enclosed by the gray box in Figure 5A) for which the prediction and error counts are nearly equal. This represents a very high error rate across a range that contains only a few positive observations, as well as a sharp discontinuity in the error rate at the threshold (Figure 5B). This suggests that the model could be improved by shifting the voting threshold to the right, i.e., to a higher tally. We have found that the error beta binomial distribution’s mean (given by α/(α + β)) provides a good alternative threshold that consistently improves specificity without undue loss of sensitivity (or vice versa, when the positive class is larger)^a^. Keeping the same networks as in model logP3-1a, but refining the model by shifting the voting threshold to the beta binomial mean, produces model logP3-1b^b^. The effect of the shift is shown in Figures 5C and D, along with the refitted error beta binomial and the refined uncertainty profile. Note that shifting the voting threshold has no effect on the distribution of predictions; which is determined by the classification thresholds for the individual networks, which do not change. The sensitivity and overall misclassification rate drop somewhat while the specificity increases (Table 1).

**Figure 6:**
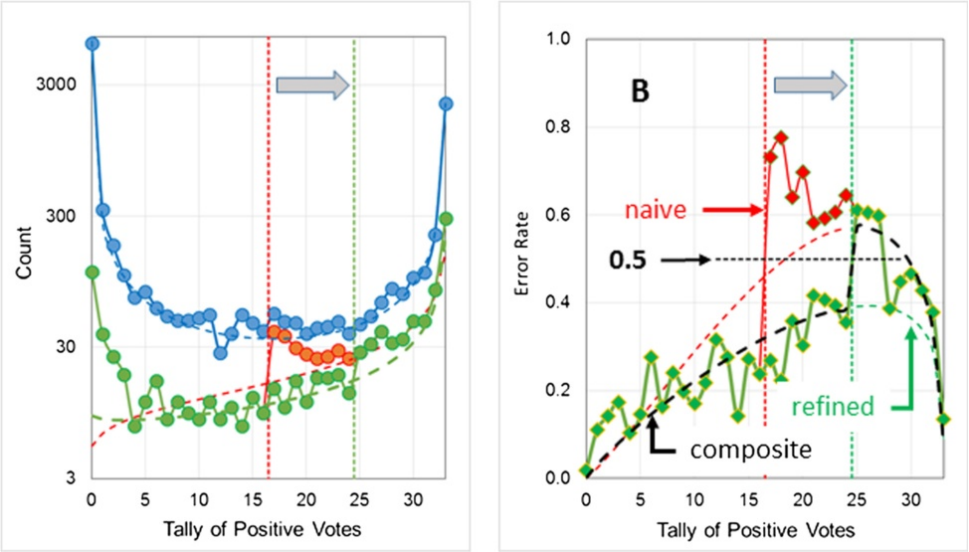
**Effect of model refinement on error distributions and uncertainty for the validation set. (A)** The error distribution for logP3-1a (for which the naive 50% voting threshold was used) is shown in red and the error distribution for logP3-1b (which was refined by shifting the voting threshold to 24.5) is shown in green. The corresponding thresholds are shown as dotted lines and the fitted beta binomials are shown as dashed lines. The distribution of predictions for both models and its fitted beta binomial are represented by the solid and dotted blue line, respectively. **(B)** The calculated uncertainties and observed error rates for models logP3-1a and logP3-1b are shown in red and green, respectively. Observed and predicted error rates are represented by solid lines and dashed lines. The black dashed line represents the composite uncertainty profile that was obtained by using the refined uncertainty profile below the threshold and the naïve uncertainty profile above it.

Figure 6 shows the effect of shifting the voting threshold on validation set performance: the fit to the errors to the left of the threshold (the false negatives) is greatly improved, and the size of the discontinuity in the error rate profile is attenuated. The naïve calculated uncertainty profile (the red dashed line in Figure 6B) is in good agreement with the probability that a given positive prediction (one to the right of the threshold) will be correct, but underestimates the confidence one can have that a negative prediction (one to the left of the threshold) is correct. On the other hand, the uncertainty estimate obtained after shifting the threshold (the green dashed line in Figure 6B) provides good estimates of the uncertainty associated with negative classifications, but underestimates somewhat the uncertainty for positive predictions that are not unanimous or nearly so. The distribution of errors between the thresholds is key to getting both curves correct, and a better error prediction profile is a composite that incorporates the discontinuity^c^. That said, the revised uncertainty profile does well enough for most purposes. The count scale in Figure 6A is logarithmic, so the number of positive predictions in the area of overestimation is usually small for unbalanced models with acceptable performance statistics.

Note that the two predicted uncertainty profiles bracket 0.5 at the refined threshold. The discontinuities near the thresholds arise because Youden’s index is used to set the classification thresholds for the individual networks. For a large training pool and a well-trained model, the density of negative predictions will be high near 0 for each network and fall off as one moves to the right, towards 1. Similarly, the density of positive predictions will be high near 1 and fall off as one moves to the left. In the limit of an infinite training pool, maximizing Youden’s index will place the classification threshold at a point where any shift to the left will increase sensitivity less than it will decrease specificity. Similarly, any increase in specificity achieved by a shift to the right will be more than offset by a decrease in sensitivity.

**Figure 7:**
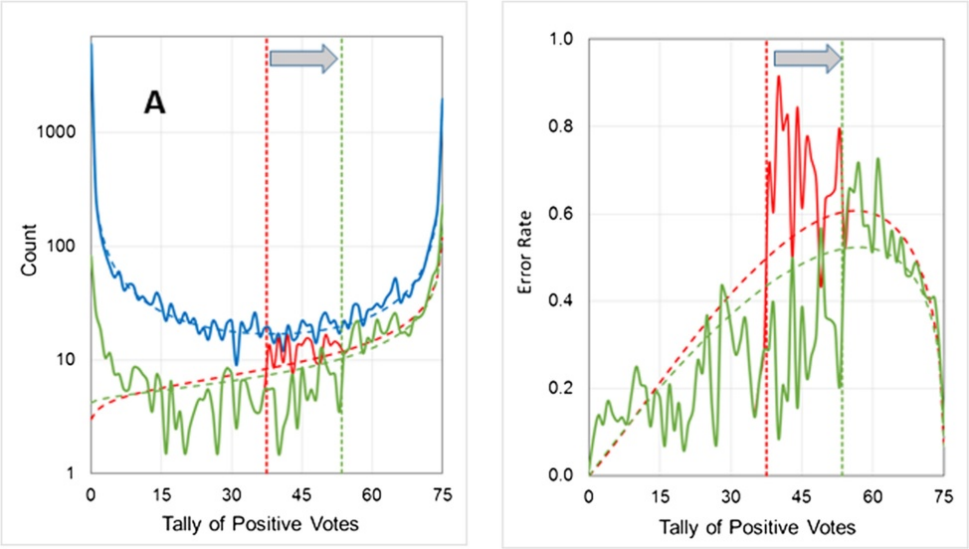
**Effect of increasing the number of networks in an ensemble.** Models logP3-2a and logP3-2b were comprised of 75 networks rather than of the default 33. Symbols have been omitted for clarity. **(A)** The validation set error distribution for logP3-2a (for which the naïve threshold of 37.5 was used) is shown in red and the error distribution for logP3-2b (refined voting threshold: 53.5) is shown in green. The corresponding thresholds are shown as vertical dotted lines and the fitted beta binomials are shown as dashed lines. The distribution of predictions and its fitted beta binomial are the same for both models; they are represented by the solid and dotted blue line, respectively. **(B)** The uncertainty profile and observed error rates for models logP3-1a and logP3-1b are shown in red and green, respectively. Observed and calculated uncertainties are represented by solid lines and dashed lines.

The 3-to-1 imbalance in the logP3 data set results in the classification threshold for each network being set at a point where the density of positive predictions is 1/3 the density of negative predictions; if the relative density were higher than that, a further shift to the left would increase *J.* Hence the error rate approaching the threshold from the left will be about 1/3 the error rate approaching from the right – once one gets close enough to it. The expected uncertainty exactly at the threshold will still be 0.5, but it will be approached from below that value from the left and from above that value from the right when the number of negatives in the populations (or the ease of their classification) exceeds the number of positives. If this rationale is correct, then the discontinuity should disappear when the network thresholds are set so as to maximize concordance instead of Youden’s index. It does indeed disappear – at the cost of a disproportionate increase in errors for the minor class (details not shown).The discontinuity is exacerbated by the compression of positive (negative) tallies as the threshold is shifted to the right (left), which can be relieved to some extent by increasing the number of networks in the ensemble. This is illustrated in Figure 7, where the number of networks was increased from 33 to 75. Note that the gap between the two predicted uncertainties is smaller than that seen in Figure 6 for a 33-network model. The error rate is somewhat underestimated at intermediate tallies – i.e., the number of errors to the left of the threshold is overestimated. The expected error counts are all below 10, however, so the residual deviation may be due to undersampling.

**Figure 8:**
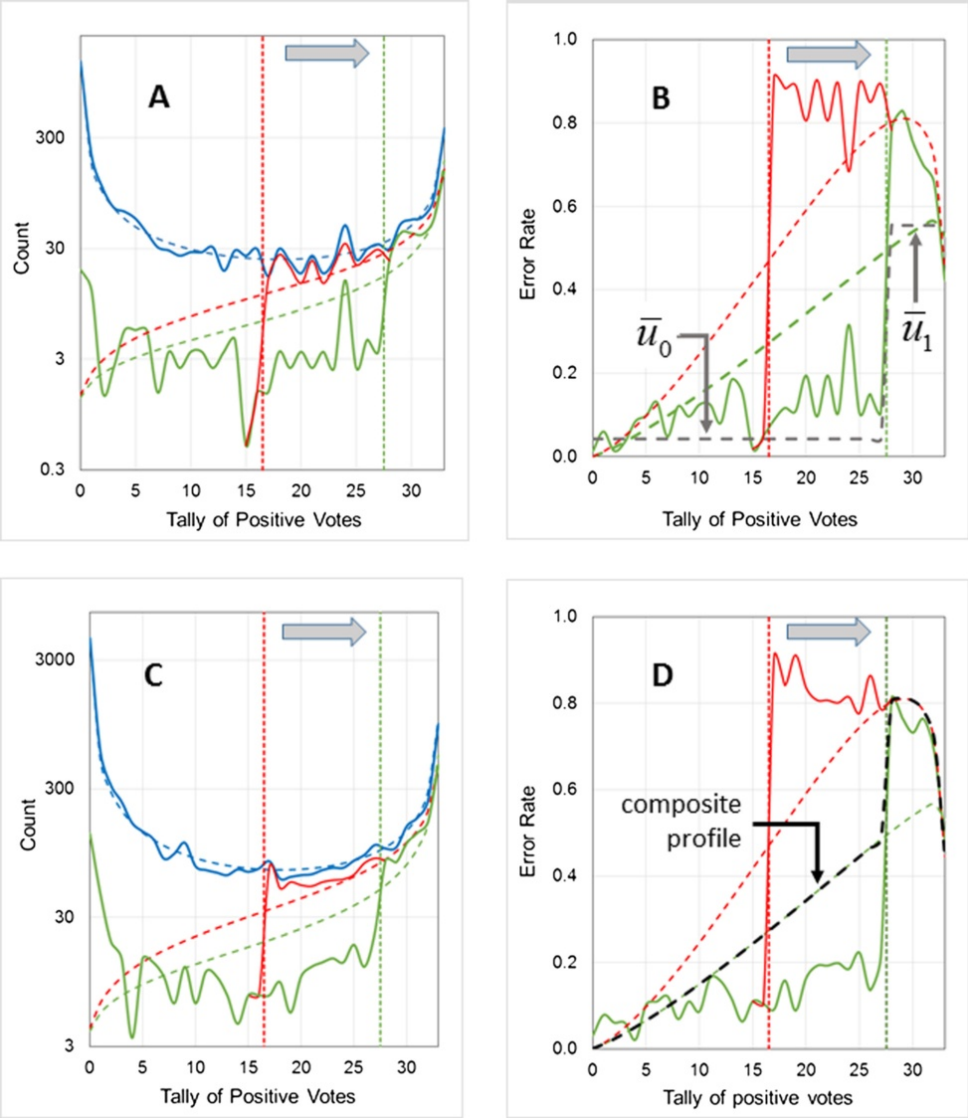
**Estimating the predictive uncertainty profile for the CYP2D6 inhibition data set.** The models shown have three hidden neurons and make use of 35 descriptors. Vertical dotted lines indicate voting thresholds. **(A)** Distribution of training pool predictions (blue), errors for model CYP2D6-1a (naïve voting threshold at 16.5; red), and errors for model CYP2D6-1b (threshold shifted to 27.5; green). Fitted beta binomial distributions are shown as dashed lines. **(B)** Calculated uncertainty profiles and distributions of training pool error rates for model CYP2D6-1a (naïve voting threshold at 16.5; red), and for model CYP2D6-1b (refined threshold at 27.5; green). Uncertainty profiles calculated from beta binomial distributions are shown as red and green dashed lines. The dashed black line indicates average class uncertainties. **(C)** Distribution of *validation set* predictions (blue), errors for model CYP2D6-1a (red), and errors for model CYP2D6-1b (green). Beta binomial distributions fitted to the *training pool* are shown as dashed lines. **(D)** Calculated uncertainty profiles and distributions of observed *validation set* error rates for model CYP2D6-1a (naïve voting threshold at 16.5; red), and errors for model CYP2D6-1b (refined threshold at 27.5; green). Uncertainty profiles calculated from beta binomial distributions fitted to the *training pools* are shown as red and green dashed lines, whereas the composite uncertainty profile is indicated by the black dashed line.

As noted earlier, the logP data sets are somewhat artificial. High-throughput screening data for CYP2D6 [29] inhibition provides an alternative, real-world example of an unbalanced data set. It is composed of 13331 observations, 1643 of which (12.3%) were categorized as “positive”. The reproducibility of this type of data is limited [30], so a larger fraction of the data set (30%) was used for model building than in the other examples discussed here.Representative results are shown in Figure 8. The uncertainties and observed errors for negative predictions by CYP2D6-1a are very low and their distributions are relatively flat: one can have high confidence that a compound predicted not to be an inhibitor truly is not. The chance of a positive prediction being a misclassification is much higher, exceeding 80% near the refined threshold and nearing 90% for the naïve threshold. This equates to a high level of false alarms, which may be tolerable if the cost of confirming a prediction (e.g., secondary screening) is acceptably low. Even for the composite uncertainty profile, however, the discrepancies for negative predictions are rather large. The plot for the training pool (Figure 8B) is very similar to that for the validation set (Figure 8D), so the limited usefulness of individual confidence estimates in this case is clear, even in the absence of a large external validation set. It is also suggested by the failure of the composite profile to bracket 0.5 at the refined threshold, though it only barely fails to do so.

Increasing the number of networks did not improve the situation in this case (details not shown). One possible alternative strategy for estimating uncertainty is to replace beta binomial profiles with average class uncertainties calculated for the training pool: ${\bar{u}}_{0}$ for the negative class and ${\bar{u}}_{1}$ for the positive class^d^. The resulting estimates are shown by the dotted gray lines in Figure 8B. As expected, the average error rates underestimate uncertainty except at the extremes of consensus, with ${\bar{u}}_{1}$ clearly inferior to the composite beta binomial estimate for positive predictions. While the latter overestimates uncertainty for negative predictions by a factor of 2–3, ${\bar{u}}_{0}$ underestimates them by a similar factor. The more conservative option – in this case, underestimating uncertainty – will be preferable in most circumstances, especially if the degree of over- and underestimation is similar.

### Confidence estimation when using averaging

It is perhaps not surprising that the uncertainty estimation method described above works well on ensemble models in which predictive classifications are determined by tallies of independently determined positive votes, since in that case the degree of network consensus reflects the variability of outcomes fairly directly. To explore how broadly applicable the method is, we turned to ANNEs in which classification is determined by averaging the individual network outputs (which are logistic functions ranging from 0 to 1 for classification models) and comparing that average to an ensemble threshold. If the average output lies above the ensemble classification threshold, the compound is classified as a positive; if the average falls below the threshold, the compound is classified as a negative. The ensemble threshold that maximizes Youden’s index across the training pool (the “*J*_max_ threshold”) is used by default. The vote tallies for estimating predictive uncertainty are obtained by comparing each network’s output to the ensemble threshold but are not themselves used for classification.

Not all networks have an equal impact on prediction, but all do contribute equally to uncertainty estimation. Those whose output lies closest to one output extreme or the other – i.e., to 0 or to 1 – shift the average most and so have the greatest “voice” in the ensemble classification; networks that dissent strongly enough from the consensus classification can sway the ensemble classification in their direction. Only in the case of unanimity does the vote tally necessarily reflect the ultimate classification: if all network outputs fall below (or above) the threshold, their average output must do so as well.

**Figure 9:**
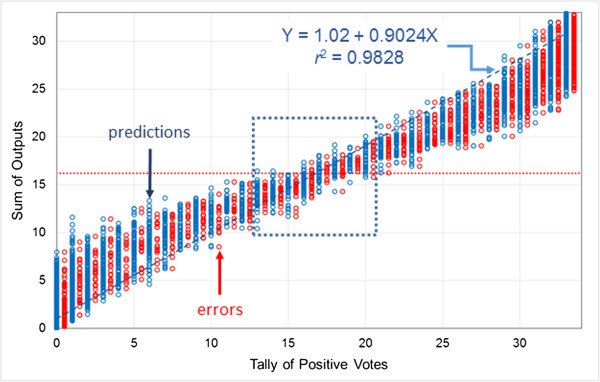
**Estimating the uncertainty profile using the averaging method.** The model whose results are shown (CYP2D6-3a) has four hidden neurons, employs 28 descriptors and uses averaging to determine ensemble classification. Blue symbols show the distribution of summed network outputs for all compounds in the validation set, whereas red symbols represent network sums for misclassified compounds; tallies for the latter are offset by 0.5 for clarity. The red horizontal dotted line indicates the classification threshold that maximizes Youden’s index *J*. The dashed line is a linear fit of all summed network outputs to the tally of positive votes. The central box highlights tallies that straddle the classification threshold for the sum.

Figure 9 shows the distribution of output sums across vote tallies for model logP3-3a, an averaging model with four hidden neurons that takes 28 descriptors as input. The *J*_max_ threshold for the average output is 0.491, corresponding to a threshold for the sum of 33 × 0.491 = 16.2 and represented by the horizontal dotted red line in Figure 9. Though the correlation between summed outputs and tallies is high for this model (*r*^2^ = 0.983), they diverge enough to spread the *J*_max_ classification threshold across several tally counts – from 13 to 20 in this case (see the central box in Figure 9).

**Figure 10:**
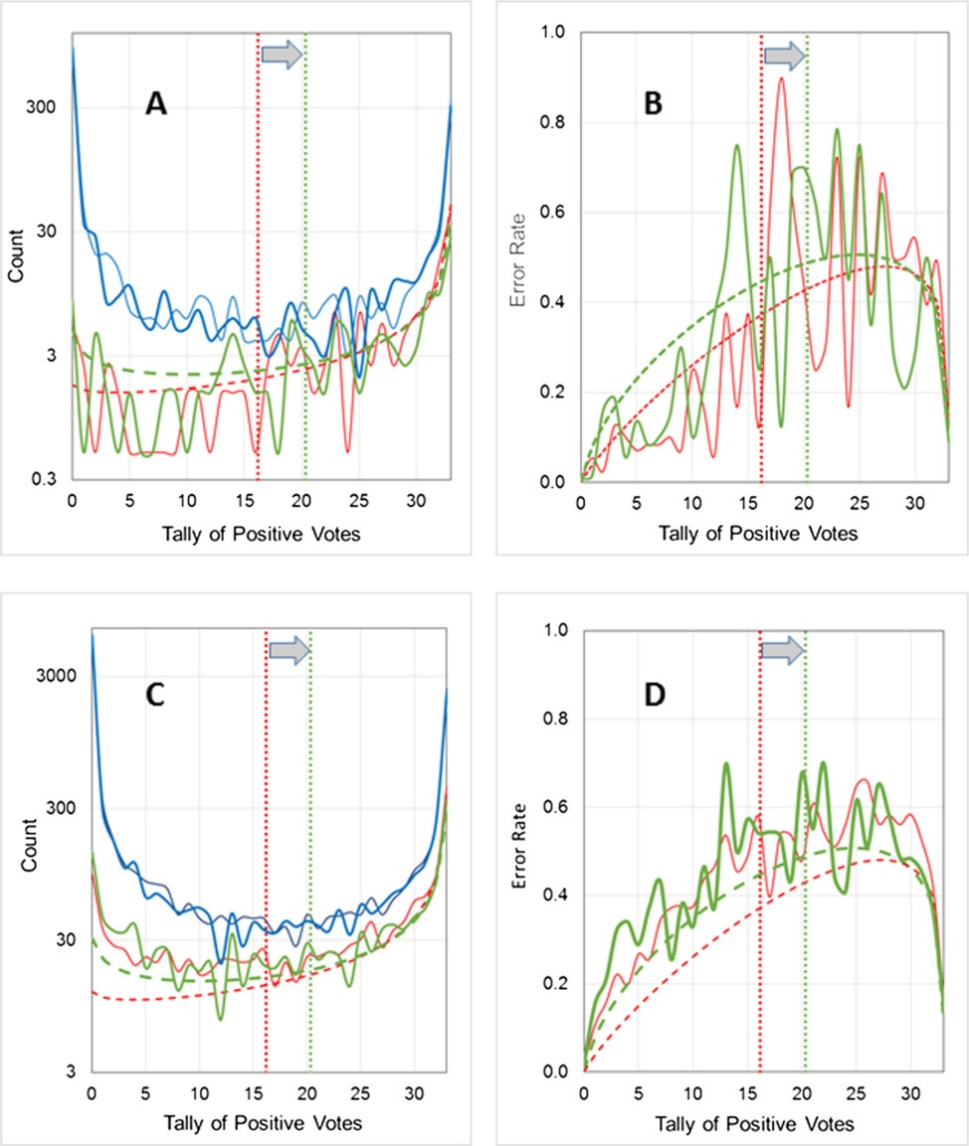
**Estimating the uncertainty profile for ensemble models constructed using the averaging method.** Thin and thick blue lines represent the distributions of predictions for logP3-3a and logP3-3b, respectively; the corresponding error and error rate distributions are shown in red and green. Uncertainty profiles and fitted beta binomials for error distributions are represented by dashed lines. Fitted prediction distributions have been omitted for clarity. Dotted vertical lines indicate thresholds for summed outputs and do not separate negative predictions and errors from positive ones (see Figure 9 and text). **(A)** Distribution of errors and predictions for the training pool. **(B)** Distribution of uncertainties and observed error rates for the training pool. **(C)** Distribution of errors and predictions for the validation set. **(D)** Distribution of uncertainties and observed error rates for the validation set.

Figure 10 shows the results of applying our beta binomial analysis to the training pool for model logP3-3a. Here, the vertical dotted red lines indicate the value (16.2) of the *J*_max_ threshold for the summed outputs. It is provided for reference only; it does not separate positive from negative predictions when averaging is used for ensemble classification.

Just as for the individual network thresholds used in the voting method, the default ensemble classification threshold used in averaging maximizes *J* and can be suboptimal in terms of predictive values. The mean of the beta binomial distribution fitted to the training pool for logP3-3a is 25.5, which is 0.772 on a per network basis; however, we have found that shifting the ensemble classification threshold to that value generally constitutes an overcorrection. Averaging models are better refined by shifting the threshold to the geometric mean of the *J*_max_ threshold and the mean of the beta binomial fitted to the initial error distribution. In this case, refinement shifted the classification threshold for the sum from 16.2 for logP3-3a to 20.3 for logP3-3b (the vertical dotted green lines in Figure 10), which is equivalent to a shift in the threshold for the average from 0.491 to 0.616.

Shifting the averaging threshold affects the entire range of tallies, not just those between the thresholds, and the distribution of predictions is affected as well as that of errors. The predictions most affected are those in which all network outputs for a particular compound lie between 0.491 and 0.616: all networks in logP3-3a will classify it as a positive, whereas all networks in logP3-3b will classify such a compound as a negative. One of those ensemble predictions will be wrong, of course, but the vote will be unanimous in both cases – a tally of 33 positive votes for logP3-3a vs. no positive votes for logP3-3b.The model using a threshold based on Youden’s index alone (logP3-3a) underestimates the error rates seen in the validation set and thereby overestimates how much confidence one can have in its individual predictions (Figure 10D). This is in contrast to model logP3-3b (indicated by green lines in Figure 10), which uses the same network outputs but a refined threshold and provides more appropriate uncertainty estimates. Here again, the less reliable model signals its weakness by having a predicted uncertainty profile that crosses its voting threshold well below 0.5. The crossover point for the model with the more robust confidence estimates, on the other hand, lies reasonably close to 0.5.Despite the unbalanced nature of the data set, the error rate profiles seen in logP3-3a and logP3-3b both lack the discontinuity near the threshold that is so evident for models built on the same data set but using the voting method to determine ensemble classification (Figure 10 vs Figure 6). This is due at least in part to the transition zone between positive and negative predictions being spread across seven tally bins rather than concentrated in two.

## Conclusions

Tallying votes and averaging submodel outputs are both useful ways to assess the degree of consensus within an ensemble. Fitting ensemble misclassification rates to a binomial distribution to assess confidence is intuitively appealing but flounders in practice because predictions by the component submodels are not independent. Using beta binomial distributions to model predictions and errors works well, however, and the ratio of such distributions can be used to estimate the likelihood that a given prediction will be incorrect.

The corresponding distributions seen in large, held-out validation sets match those seen for the training pool remarkably well in most cases. Moreover, those cases for which the predicted error rate profile is not reliable provide a good clue to that effect, in that their calculated uncertainty profiles cross the model’s voting threshold (or, in the case of averaging, the ensemble classification threshold applied to the summed outputs) well below the expected value of 0.5. Why this occurs is not clear at this time but it may reflect a lack of diversity in predictions among the networks that make up the ensemble. If consensus is complete across the entire training pool, for example, all predictions and errors will lie at the extremes and no information will be available regarding the distribution of predictions and errors in between.

Models based on voting that are built on unbalanced data sets and use Youden’s index as the criterion for setting individual network classification thresholds tend to have a discontinuity in observed error rates near the ensemble voting threshold. Resetting that threshold to match the mean of the beta binomial for the error, ϵ(*k*), helps attenuate that discontinuity. In many cases, the uncertainty profile can still be adequately described by a combination of just two distributions – one fitted to the training pool predictions and another fitted to the training pool errors. In other cases, four sets of distribution parameters and a threshold value are required to create a composite distribution, one pair applying to the left of the threshold (where the true and false negatives are found) and the other pair to its right (where true and false positives are found). Models built using the averaging method avoid this complication.

Though the examples discussed here all involve ANNE classification models, the technique should be applicable to any ensemble classification model in which the constituent submodels represent subsamples of a shared model space and the predictions for individual submodels are accessible. Random forest models come to mind as one example, but most methods that involve bagging [31] probably also qualify. Most importantly, perhaps, the training pool distributions should be equally applicable to external predictions – provided that, as is the case in these examples, model building is done in a way that avoids overtraining and the training pool is representative of the population for which uncertainty estimates are desired. If the model-building tool used requires an artificially biased training pool, it needs to be augmented with examples from the undersampled class before the prediction and error beta binomials are fitted. Applying this approach to the logP3 data set yields results qualitatively similar to those seen when using an unbalanced training pool (details not shown), but full validation of such an approach is beyond the scope of this paper.

Our approach complements classical overall performance measures based on partitioning predictions into true positive, false positive, true negative and false negative categories; it does not replace them. Implementing it is straightforward: it was originally done using the GAMMALN function and the Solver add-in for Excel [32] to fit the beta binomial parameters. The uncertainty values obtained thereby have subsequently been incorporated into many of the models distributed with ADMET Predictor 7.0 as predictive “confidences” (equal to 1 – *u*(*k*)). Such confidence values can also be generated for classification models created using the ADMET Modeler module of the program.

## Experimental

### Data sets

The log P data set is diverse and has been heavily curated. It consists of the 12,580 values used to build and test the S + logP model distributed with ADMET Predictor, the bulk of which are derived from the BioByte database [33]. Some entries have been modified to accommodate discrepancies found with respect to literature references, while others have been added from the original literature in the interests of expanded coverage of chemistry and property space. Categorizing compounds having log P ≥ 2.0 as “positive” and those having log P < 2.0 as “negative” yielded a relatively balanced data set (logP2) comprised of 5946 positives and 6634 negatives.

The Ames mutagenicity data set was taken from the compilation by Hansen *et al.*[26] and contains data of medium quality. A handful of structures were corrected for structural errors and redundant entries were removed, as were salts of metals other than sodium or potassium. It is quite a balanced data set: of the 6471 entries surviving curation, 2983 (46%) were categorized as “positives” based on their having been classified as “active” in the original publication.

High-throughput screening data on CYP2D6 inhibition comes from PubChem AID 1851 [29,34] provided an example of an unbalanced real-world data set. Here the negative class was comprised of compounds for which no significant inhibition was seen at any concentration of the test compound (“activity outcome” = 1 [34]) or for which the AC50 was ≥ 10 μM. The positive class was made up of compounds for which the AC50 was < 10 μM.

An indication of titration reliability was provided for each compound and those labeled as reflecting dubious titrations (“activity outcomes” of 3 [34]) were disregarded. There were a substantial number of such unreliable titrations: 35% of the positives and 70% of the negatives for which an AC50 was obtained. Salts were split into their constituent components and the largest component based on number of atoms was retained. Organometallics were eliminated and replicated evaluations [30] were resolved by majority rule of their reliable titrations. In cases where the number of valid positive and negative entries were equal, the structure was discarded. This left a data set of 13331 observations, 1643 of which (12.3%) were categorized as “positive”. The majority (70%) of the observations were selected at random and set aside as a validation set comprised of 1158 positives and 8180 negatives. The remaining 3993 observations were used for model training and ensemble selection.

Tautomeric ambiguities for all data set entries were resolved using the pK_a_ predictions and microstate analysis in ADMET Predictor 7.0.

## Methods

Models considered here were ensembles of 33 artificial neural networks (except as otherwise noted) constructed in the ADMET Modeler module of a prerelease version of ADMET Predictor 7.0, with all the networks in a given ensemble having an identical architecture, i.e., the same set of descriptor inputs and number of hidden neurons. We do not expect that the conclusions drawn here are limited in applicability by exactly how the particular ensemble models were constructed, but the general procedure followed to build them is described in some detail below.

### Model construction

Data remaining after extraction of the external validation set was further divided into a training pool (further divided into “training” and “verification” sets) and an external test set. The latter is used to help identify the ensemble having the “best” architecture but is not used during the model building process itself. A grid of ensemble architectures is trained using a total of 165 networks per architecture from which the best 33 networks are selected. Each architecture uses a different combination of neurons and inputs and each network has its own training and verification set. Two thirds of the training pool was used for training and the remaining third served as a verification set. These training pool splits were made randomly and independently for each network in the ensemble.

Each input (descriptor) *x*_*i*_ is assigned a weight *w*_ij_ for each neuron *j* in the single hidden layer such that the output *f*_j_ for neuron *j* is a function of the input values as given by:

(6) $$f_{j}=\tanh\left(\sum_{i}w_{\mathrm{ij}}x_{i}-t_{j}\right)$$

where *t*_j_ is the offset associated with neuron *j*. Those neural outputs are combined using an output function g defined by:

(7) $$g=\mathrm{lg}\left(\sum_{j}a_{j}f_{j}-b\right)$$

where “lg” represents the logistic function, *b* is a network offset, and *a*_j_ is a weight associated with neuron *j* in the hidden layer. The adjustable parameters *w*_ij_, *t*_j_, *a*_j_ and *b* for each network are initialized independently with random values, then adjusted iteratively to minimize the objective function *Obj*:

(8) $$\mathrm{Obj}=\sum_{l=1}^{n}c_{0}\left(1-q\left(l\right)\right){\left(g\left(l\right)\right)}^{2}+c_{1}q\left(l\right){\left(1-g\left(l\right)\right)}^{2}$$

where *q*(*l*) is the class indicator value for observation *l* (0 for negatives and 1 for positives) and *g*(*l*) is the output function evaluated on the input vector **x**_*l*_. The net effect of minimizing *Obj* is to drive the outputs for observations in the negative and positive classes towards 0 and 1, respectively. The class weights *c*_0_ and *c*_1_ are the fractions of observations in the positive and negative classes, respectively, resulting in the smaller class having the larger weight.

The verification set is used for early stopping [35], which reduces the tendency to overtrain. The objective function is evaluated against the verification set after each iterative incremental improvement with respect to the training set and network training is halted when the objective function for the verification set fails to improve for 15 consecutive iterations. A classification threshold is then determined for each network that maximizes Youden’s index *J* for the training set:

(9) J=sensitivity+specificity–1

Unless otherwise indicated, 165 networks were trained for each architecture (number of inputs and number of neurons) and the 33 having the best performance (combining training and verification statistics) were retained in the final ANNE for that architecture. A matrix of architectures is trained, varying the numbers of inputs and neurons, and the architecture that provides the best, statistically significant performance is selected as the final model.

ADMET Modeler combines outputs from the constituent networks in an ensemble model in one of two ways: one is to tally positive “votes” based on each individual network’s own threshold (see above), whereas the other is to compare the averaged output to an ensemble threshold that maximizes *J* for the average. The former is the “voting method”, whereas the latter is the “averaging method”.

Candidate ensembles are compared on the basis of their performance on the training and test sets that they share. To ensure that the results obtained are qualitatively robust, experiments should be run across a range of training pool/test set splits, a range of architectures and a range of random number seeds – as was done for the present study.

### Confidence estimation

The distributions *f* and *e* of predictions and predictive errors, respectively, are calculated as functions of the number *k* of positive votes for compounds in the training pool, which ranges from 0 to *K* (the number of networks in the ensemble; *K* = 33 unless otherwise noted). A continuity correction [25] of 0.5 error or 1 prediction is added to the respective counts for each value of *k* to help compensate for undersampling (see above). As a result, the observed contingent error rate is *e*(*k*)/*f*(*k*) = 0.5 for tallies with no predictions, 0.5/2 = 0.25 for tallies having a single correct prediction, and 1.5/2 = 0.75 for tallies with a single incorrect prediction.

Beta binomial distributions ϵ(*k*) and *φ*(*k*) are fitted to the cumulative observed error and prediction distributions, respectively. Intermediate tallies away from the extremes tend to be sparsely sampled even for quite large data sets, however, which can complicate direct fitting. This is addressed in part by the continuity correction described above and in part by fitting cumulative beta binomials to the respective cumulative observed distributions by minimizing the Kolmogorov-Smirnov statistic [21] rather than carrying out a direct fit for the corresponding density functions. The probability that a prediction having *k* positive votes is in error (the uncertainty) is then given by:

(10) $$u\left(k\right)\;=\mathrm{MR}*\epsilon \left(k\right)/\varphi \left(k\right)$$

where *MR* is the overall misclassification rate for the training pool. Confidence in the prediction is reported as 1 – *u*(*k*). In the case where the confidences are the same for all compounds of a given class, this reduces to the positive or negative predictive values for the respective classes.

### Descriptors

Of the 366 molecular descriptors generated by default in ADMET Modeler, the analyses presented here used a subset of 221: substructure counts (e.g., nitro, amide, ester groups, etc.) were omitted to maximize the size of the applicability domain, as were molecular weight, total bond count, number of hydrogens and four other descriptors. The latter group was dropped because they were highly correlated with more informative ones. Other descriptors were set aside for individual analyses if they had a coefficient of variation less than 1%, failed to be nonzero in at least 4 cases, or had absolute correlation coefficients with one or more other descriptors above 0.98. The number of descriptors passing those filters in each case ranged from 172 to 183 and included: counts of common element and bond types, rings and molecular volumes; electrotopological and connectivity indices; topological size measures; molecular and partial atomic charge values; polarizabilities; and topological autocorrelation vectors of various atomic properties such as partial charge and Fukui indices.

The ranking of descriptors was determined using the Input Gradient option in ADMET Modeler, wherein candidate descriptors are ranked by analysis of their response gradients. Trial neural networks with a specified number of hidden neurons (here, 1 to 6) are built using all descriptors, then the analytical sensitivity gradient is extracted from that network for each candidate descriptor. Then, a series of ensemble models are built for a given number of neurons in which the number of descriptors is progressively increased, selecting them based on the ranking assigned by the Input Gradient procedure. The resulting Input Gradient descriptor rankings are thus dependent on the number of neurons used.

## Endnotes

^a^The means for the error beta binomials for the logP2 and Hansen data sets are very close to 16.5, and shifting the voting threshold has little effect.

^b^Models whose names differ only by a terminal letter have the same component networks but have different classification criteria at the ensemble level.

^c^To avoid ties, the actual threshold is calculated from the theoretical threshold set to floor(mean)+0.5.

^d^Note that ${\bar{u}}_{0}$ = 1 – (negative predictive value) and ${\bar{u}}_{1}$ = 1 – (positive predictive value).

## Competing interests

The authors have no competing interests apart from their institutional affiliation with Simulations Plus, Inc.

## Authors’ contributions

All authors contributed to the manuscript and helped carry out the extensive testing needed to make application of the method it describes practical and robust. In addition, MW derived the formalism for estimating the contingent uncertainty profile from the observed distributions of predictions and errors and implemented it in Excel. WL refined the Excel implementation and encoded it in C++ for use in ADMET Predictor under RF’s supervision. RDC recognized the suitability of beta binomial distributions and the need to apply continuity corrections. ACL and MSL carried out preliminary analyses demonstrating the usefulness of the log P data set for investigating the distributions of classification error and the suitability of the binomial distribution. All authors read and approved the final manuscript.

## References

[B1] Eriksson L Jaworska J Worth AP Cronin MTD McDowell RM Gramatica P Methods for reliability and uncertainty assessment and for applicability evaluations of classification- and regression-based QSARs Environ Health Perspect 2003 111 1361 1375 12896860 10.1289/ehp.5758 PMC1241620

[B2] Worth AP Puzyn T, Leszczynski J, Cronin MT The role of QSAR methodology in the regulatory assessment of chemicals Advances in Computational Chemistry and Physics Volume 8: Recent Advances in QSAR Studies 2010 Netherlands: Springer 367

[B3] Sahlin U Uncertainty in QSAR Predictions Altern Lab Anim 2013 41 111 125 23614548 10.1177/026119291304100111

[B4] Weaver S Gleeson MP The importance of the domain of applicability in QSAR modeling J Mol Graph Model 2008 26 1315 1326 18328754 10.1016/j.jmgm.2008.01.002

[B5] Tong W Xie Q Hong H Shi L Fang H Perkins R Assessment of prediction confidence and domain extrapolation of two structure–activity relationship models for predicting estrogen receptor binding activity Environ Health Perspect 2004 112 1249 1254 15345371 10.1289/txg.7125 PMC1277118

[B6] Tetko IV Sushko I Pandey AK Zhu H Tropsha A Papa E Öberg T Todeschini R Fourches D Varnek A Critical assessment of QSAR models of environmental toxicity against *Tetrahymena pyriformis*: Focusing on applicability domain and overfitting by variable selection J Chem Inf Model 2008 48 1733 1746 18729318 10.1021/ci800151m

[B7] Beck B Breindl A Clark T QM/NN QSPR models with error estimation: Vapor pressure and logP J Chem Inf Comput Sci 2000 40 1046 1051 10955536 10.1021/ci990131n

[B8] Clark RD DPRESS: localizing estimates of predictive uncertainty J Cheminform 2009 1 11 20298517 10.1186/1758-2946-1-11 PMC3225832

[B9] Sahlin U Jeliazkova N Öberg T Applicability domain dependent predictive uncertainty in QSAR regressions Mol Inf 2011 30 551 564 10.1002/minf.201200131 27485196

[B10] Wood DJ Carlsson L Eklund M Norinder U Stålring J QSAR with experimental and predictive distributions: an information theoretic approach for assessing model quality J Comput Aided Mol Des 2013 27 203 219 23504478 10.1007/s10822-013-9639-5 PMC3639359

[B11] Keefer CE Kauffman GW Gupta RR Interpretable, probability-based confidence metric for continuous quantitative structure–activity relationship models J Chem Inf Model 2013 53 368 383 23343412 10.1021/ci300554t

[B12] Sheridan RP Using random forest to model the domain applicability of another random forest model J Chem Inf Model 2013 53 2837 2850 24152204 10.1021/ci400482e

[B13] Sahlin U Jeliazkova N Öberg T Applicability domain dependent predictive uncertainty in QSAR regressions Mol Inf 2014 33 26 35 10.1002/minf.201200131 27485196

[B14] Sushko I Novotarskyi S Körner R Pandey AK Kovalishyn VV Prokopenko VV Tetko IV Applicability domain for *in silico* models to achieve accuracy of experimental measurements J Chemometrics 2010 24 202 208

[B15] Sushko I Novotarskyi S Körner R Pandey AK Cherkasov A Li J Gramatica P Hansen K Schroeter T Klaus-Robert Müller K-R Xi L Liu H Yao X Öberg T Hormozdiari F Dao P Sahinalp C Todeschini R Polishchuk P Artemenko A Kuz’min V Martin TM Douglas MDM Fourches D Muratov E Tropsha A Baskin I Horvath D Marcou G Muller C Applicability domains for classification problems: benchmarking of distance to models for Ames mutagenicity set J Chem Inf Model 2010 50 2094 2111 21033656 10.1021/ci100253r

[B16] Bodor N Harget A Huang M-J Neural network studies.1. Estimation of the aqueous solubility of organic compounds J Am Chem Soc 1991 113 9480 9483

[B17] Lindsey JK Response surfaces for overdispersion in the study of the conditions for fish eggs hatching Biometrics 1999 55 149 155 11318149 10.1111/j.0006-341x.1999.00149.x

[B18] Dávila E López LA Díaz LG A statistical model for analyzing interdependent complex of plant pathogens Rev Colomb Estad 2012 35 255 270

[B19] Moon H Ahn H Kodell RL Baek S Lin C-J Lee T Chen JJ **Ensemble methods for classification of patients for personalized medicine with high-dimensional data** Artif Intell Med 2007 41 197 207 17719213 10.1016/j.artmed.2007.07.003

[B20] Abramowitz M, Stegun IA Handbook of Mathematical Functions with Formulas, Graphs, and Mathematical Tables, Tenth Printing 1972 Washington: National Bureau of Standards

[B21] Massey FJ Jr The Kolmogorov-Smirnov test for goodness of fit J Am Stat Assoc 1951 46 68 78

[B22] Simulations Plus, Inc ADMET Predictor™ [http://www.simulations-plus.com]

[B23] Dearden JC Netzeva TI Bibby R Ford M, Livingstone D, Dearden J, Van de Waterbeemd H A comparison of commercially available software for the prediction of partition coefficient EuroQSAR 2002: Designing Drugs and Crop Protectants: Processes, Problems and Solutions 2003 Oxford: Blackwell Publishing 168 169

[B24] Mannhold R Poda GI Ostermann C Tetko IV Calculation of molecular lipophilicity: State-of-the-art and comparison of log P methods on more than 96,000 compounds J Pharm Sci 2009 98 861 893 18683876 10.1002/jps.21494

[B25] Sweeting MJ Sutton AJ Lambert PC What to add to nothing? Use and avoidance of continuity corrections in meta-analysis of sparse data Stat Med 2004 23 1351 1375 15116347 10.1002/sim.1761

[B26] Hansen K Sebastian Mika S Schroeter T Sutter A Ter Laak A Steger-Hartmann T Heinrich N Müller K-R Benchmark data set for *in silico* prediction of Ames mutagenicity J Chem Inf Model 2009 49 2077 2081 19702240 10.1021/ci900161g

[B27] Benigni R Giuliani A Computer-assisted analysis of interlaboratory Ames test variability J Toxicol Environ Health 1988 25 135 148 3418743 10.1080/15287398809531194

[B28] Youden WJ Index for rating diagnostic tests Cancer 1950 3 32 35 15405679 10.1002/1097-0142(1950)3:1<32::aid-cncr2820030106>3.0.co;2-3

[B29] National Institutes of Health AID 1851 – PubChem BioAssay Summary PubChem Bioassay 1851 [http://pubchem.ncbi.nlm.nih.gov/assay/assay.cgi?aid=1851; accessed 10 July 2013]

[B30] Sun H Veith H Xia M Austin CP Tice RR Huang R Prediction of cytochrome P450 profiles of environmental chemicals with QSAR Models built from drug-like molecules Mol Inform 2012 31 783 792 23459712 10.1002/minf.201200065 PMC3583379

[B31] Breiman L Bagging predictors Mach Learn 1996 24 123 140

[B32] Harris DC Nonlinear least-squares curve fitting with microsoft excel solver J Chem Ed 1998 75 119 121

[B33] BioByte Corp BioByte Master Database [http://www.biobyte.com]

[B34] Veith H Southall N Huang R James T Fayne D Artemenko N Shen M Inglese J Austin CP Lloyd DG Auld DS Comprehensive characterization of cytochrome P450 isozyme selectivity across chemical libraries Nat Biotechnol 2009 27 1050 1055 19855396 10.1038/nbt.1581 PMC2783980

[B35] Yan A Gasteiger J Prediction of aqueous solubility of organic compounds based on a 3D structure representation J Chem Inf Comput Sci 2003 43 429 434 12653505 10.1021/ci025590u

